# Plasminogen activator inhibitor-1 concentrations and bone mineral density in postmenopausal women with type 2 diabetes mellitus

**DOI:** 10.1186/s12902-016-0094-x

**Published:** 2016-03-03

**Authors:** Silvija Canecki-Varžić, Ivana Prpić-Križevac, Ines Bilić-Ćurčić

**Affiliations:** Department of Diabetes, Endocrinology and Metabolism Disorders, University Hospital Center Osijek, Faculty of Medicine, University of Osijek, J. Huttlera 4, HR-31000 Osijek, Croatia

**Keywords:** Diabetes type 2, Plasminogen activator inhibitor-1, Bone mineral density, Bone turnover markers, Osteoporosis, Osteopenia

## Abstract

**Background:**

Women with type 2 diabetes mellitus (T2DM) have a higher risk of fractures despite increased bone mineral density (BMD). In experimental studies a potential role of plasminogen activator inhibitor-1 (PAI-1) in bone remodeling is suggested but studies in humans are lacking. This is a first study in humans investigating whether circulated levels of PAI-1 in postmenopausal women with T2DM are related to BMD and adiposity.

**Methods:**

Anthropometric variables, PAI-1 and insulin levels, serum lipids and bone turnover markers were measured in 127 postmenopausal women with T2DM. A total of 117 female patients were divided according to lumbar spine BMD measurements via dual-energy x-ray absorptiometry in three groups: 47 with osteopenia, 21 with osteoporosis and 49 with normal BMD.

**Results:**

Diabetic patients with normal BMD had significantly higher BMI, greater waist circumference and lower bone turnover markers than diabetics with osteopenia and osteoporosis. PAI-1 was lower in diabetics with osteoporosis and osteopenia compared with diabetics with normal BMD. Multiple regression analysis revealed insulin, triglycerides levels, pyrilinks and beta blocker therapy to be the strongest predictors of PAI-1 levels. PAI-1 levels correlated with both L-BMD and hip BMD, but after adjustment for age and BMI association was no longer significant.

**Conclusion:**

Our findings suggest that elevated PAI-1 levels are associated with higher BMD in obese diabetic patients but the possible implications of this finding and underlying mechanisms still remain unclear. Obviously, metabolic parameters, may affect both BMD and PAI-levels, and association of PAI-1 and BMD could be indirect. However, as pyrilinks is also independently and significantly negatively correlated to PAI-1 its direct involvement in bone metabolism is also plausible. Further investigations are needed to elucidate the nature of interaction of this matrix modulator in relation to energy and bone metabolism in humans.

**Electronic supplementary material:**

The online version of this article (doi:10.1186/s12902-016-0094-x) contains supplementary material, which is available to authorized users.

## Background

Diabetes mellitus type 2 (T2DM) could influence bone through several mechanisms, some of which may have contradictory effects. Traditionally it is thought that obesity or high body mass index (BMI) has a beneficial effect on bone mass thus protecting against excessive bone loss in aging [1, 2]. This is probably a result of enhanced osteoblast differentiation due to heavier mechanical loading of bone, whereas hyperinsulinemia may promote bone formation [3]. However, previous studies have suggested that bone quality is poor in diabetic patients causing impaired fracture healing, but underlying mechanisms remain unclear. One of the possibilities is increased resorption or suppressed bone turnover mediated through low-grade inflammation present in diabetes type 2 [4, 5]. One of those proinflammatory cytokines is plasminogen activator inhibitor-1 (PAI-1) and its increased levels are present in patients with diabetes type 2 and metabolic syndrome [6, 7], while adipose tissue is a significant determinant of PAI plasma concentration [8–10]. Furthermore, increased PAI-1 activity has been associated with polycystic ovarian syndrome (PCOS) as well as with the progression of atherosclerosis [11]. Also, PAI is a recognized modulator of matrix and could be implicated in the bone homeostasis through regulation of bone size during developmental growth and determination of fracture callus size, cartilage formation, and resorption during bone fracture repair as was shown in murine model [12, 13]. Obviously, there is growing evidence of the importance of PAI in bone metabolism, however studies in humans are lacking in particular in patients with osteoporosis. The aim of this study was to investigate whether circulated levels of PAI-1 in postmenopausal women with T2DM are related to BMD and adiposity. To our knowledge this is a first study performed on human subjects with osteoporosis and diabetes type 2 including patients with long-term diabetes who have received standard care according to American Diabetes Association (ADA) guidelines [14], thus all medications were carefully recorded and the possible role of the subsequent modification of inflammation/coagulation was investigated.

## Methods

### Study design

This study complied with the Declaration of Helsinki and was approved by ethics committee of University Hospital Center Osijek. Informed and written consent was obtained from all participants. The subjects of this study were a subset of all patients who visited the outpatient clinic for a regular medical check-up or were hospitalized because of poor metabolic control at the Department of Endocrinology and Diabetes, Clinical Hospital Center Osijek. In this cross sectional study a total of 117 postmenopausal female patients with diabetes type 2 were included and then divided according to lumbar spine BMD (LBMD) measurements via dual-energy x-ray absorptiometry (DXA) in three groups: 47 with osteopenia (T-score between −1 and −2,5), 21 with osteoporosis (T-score ≤ −2,5) and 49 with normal BMD (T-score of −1 or higher).

### Patients

Inclusion citeria were as follows: female gender, diabetes mellitus type 2 and menopause. Patients with a history of the following conditions were excluded from the study: those taking medication for osteoporosis; those receiving medications affecting BMD, such as glucocorticoids; those with conditions, such as malignancy, thyroid disease or thyroid functional abnormality, that would be apparent causes for abnormal bone mass and those with acute infectious disease or chronic inflammatory diseases.

At the time of inclusion detailed medical history was obtained, physical exam and ECG was performed. Each patient underwent DXA for assessment of bone mineral density and BMI was calculated from body weight and height (kg/m^2^). Waist circumference at the bending point was measured in centimeters with the participant wearing only a single layer of clothes. Postmenopausal status was defined, through a close history taking, as cessation of menstruation for at least 1 year.

### BMD measurement

The BMD of the lumbar spine and total hip was measured by dual energy X-ray absorptiometry (Dexa QDR 1000, Hologic, Inc., Waltham, MA) calibrated daily using a standard phantom provided by the manufacturer. Lumbar spine BMD was the mean of lumbar vertebrae 1–4.

### Biochemical assay

At study entry, all women underwent blood analyses to verify a healthy state. All blood samples, incuding samples for determing bone turnover markers, were obtained after overnight fast. Blood samples were drawn to determine routine laboratory tests including cholesterol, triglycerides, HDL-C, LDL-C, urea, creatinine, HbA1c. Measurements of plasma glucose, HbA1c, lipids, renal function and creatinine clearance were performed by routine assays using automatic analyzer Olympus AV 640 (Olympus, Beckman Coulter, Inc). Insulin levels were measured by commercial available radio-immunoassay (RIA) kit (SorinBiomedica, Italy) according to manufacturer’s recommendations. Bone metabolism was evaluated by determining the serum levels of osteocalcin (OC) as marker of bone formation, and urinary pyrilinks-D (PYD) and beta-CrossLaps (B-CTx), as markers of bone resorption. Second morning urine sample was collected and PYD in urine was measured using competitive enzyme immunoanalysis by commercially available kit, according to Manufacturer’s protocol (Metra Biosystem INC., Mountain View, SAD, reference value 3.0–7.4 nM DPD/mM creatinine). Serum OC levels were assayed by an immunoradiometric assay (IRMA) with commercially available kits, according to manufacturer’s recommendations (BioSource Europe S.A., Nivelles, Belgium, reference value 5–25 ng/mL). Serum Beta-CTx was measured by electrochemiluminescent immunoassay (ECLIA) by commercially available kit, according to Manufacturer’s protocol (Roche-B-Crosslaps assay). Fibrinogen and plasminogen activator inhibitor-1 (PAI-1) were determined by coagulometry using automated photometric coagulation analyzer BCS (Behring Coagulation System) X5.

### Statistical analysis

Continuous variables were summarized using mean and standard deviation values. Significance was declared at a two-sided 0.05 level, unless otherwise specified. Mann–Whitney test or parametric *t*-test was used for between group comparisons. Analysis of variance (ANOVA) was used to determine the significance of difference between three groups. For data which was not normally distributed analysis of variance was performed with Kruskal Wallis ANOVA. Relationships between variables were analyzed using Spearman’s coefficients of correlations. Stepwise multiple regression analysis with forward selection was used to examine the relationships among variables. Results of regression analysis were given as beta coefficients and R^2^. All statistical analysis was performed using the program package StatSoft, Statistica12.0.

## Results

### Characteristics of diabetic postmenopausal women with normal BMD compared to group with decreased BMD

Baseline characteristics of the subjects are summarized in Table 1. All groups were similar in the diabetes duration and HbA1c levels. A total of 117 diabetic patients were divided according to L-BMD measurements in three groups: 47 with osteopenia (T-score between −1 to −2,5), 21 with osteoporosis (T-score ≤ −2,5) and 49 with normal BMD (T-score ≥ −1). Groups were matched according to age, smoking, alcohol consumption, antihypertensive and statin therapy as well as hormone replacement therapy and oral antidiabetic drugs (Additional file 1: Table S1). No one received pioglitazone nor SGLT2 inhibitors. Diabetic patients with normal BMD had significantly higher BMI (*p* = 0,02; *p* = 0,002 respectively) and greater waist circumference (*p* = 0,03; *p* = 0,003 respectively) than diabetics with osteopenia and osteoporosis. They had shorter menopause period (*p* = 0,049) and slightly but significantly lower LDL cholesterol (*p* = 0,042) compared with diabetics with osteoporosis. When compared to both groups with bone loss, diabetics with normal BMD had significantly lower bone turnover markers (*p* < 0,01). PAI-1 was the lowest in diabetic patients with osteoporosis (*p* < 0,05) and slightly but not significantly lower in patients with osteopenia (*p* < 0,052) compared with diabetics with normal BMD.

**Table 1:** Baseline characteristics of postmenopausal women with T2DM

	Osteoporosis *n* = 21	Osteopenia *n* = 47	Normal BMD *n* = 49	*p*
Age (year)	65,3 ± 10,2	67,4 ± 8,04	64,9 ± 8,7	NS
Waist (cm)	89,8 ± 11,2	96,0 ± 8,2	98,3 ± 12,0	^c^ **0,03;** ^b^ **0,003**
BMI (kg/m^2^)	27,5 ± 5,6	29,1 ± 3,9	31,3 ± 5,1	^a^ **0,02;** ^b^ **0,002**
Menopause duration (year)	17,8 ± 9,2	18,3 ± 8,5	14,7 ± 7,9	^a^ **0,049**
Diabetes duration (year)	9,1 ± 6,3	12,2 ± 7,1	12,1 ± 7,3	NS
HbA1c (%)	10,2 ± 2,24	10,1 ± 1,59	9,95 ± 1,9	NS
Cholesterol (mmol/l)	5,45 ± 1,62	5,13 ± 1,25	4,97 ± 1,41	NS
HDL cholesterol (mmol/l)	1,29 ± 0,25	1,22 ± 0,25	1,19 ± 0,27	NS
LDL cholesterol (mmol/l)	3,39 ± 1,02	3,03 ± 1,02	2,76 ± 1,12	^ab^ **0,042**
Triglicerydes (mmol/l)	1,98 ± 1,32	1,96 ± 0,79	2,37 ± 1,43	NS
Osteocalcin (ng/ml)	10,22 ± 7,67	8,37 ± 3,84	6,32 ± 2,87	^a^ **0,025;** ^b^ **0,0013**
Insulin (mIU/l)	8,94 ± 4,14	9,97 ± 4,82	10,62 ± 5,69	NS
Pyrilinks (nMPD/mMc)	7,8 ± 3,1	7,41 ± 2,9	5,5 ± 2,1	^a^ **0,001;** ^b^ **0,0015**
Crosslaps (ng/ml)	0,54 ± 0,25	0,49 ± 0,29	0,35 ± 0,21	^a^ **0,012;** ^b^ **0,0092**
PAI-1 (U/l)	3,3 ± 2,1	3,5 ± 2,1	4,4 ± 1,9	^a^ **0,042**; ^b^0,052
Fibrinogen (g/L)	4,1 ± 1,0	4,3 ± 0,8	4,0 ± 0,8	NS
CRP (mg/L)	5,77 ± 11,6	3,89 ± 3,67	3,74 ± 4,25	NS
Lumbar BMD (g/cm^2^)	0,707 ± 0,07	0,856 ± 0,04	1,036 ± 0,09	^ab^ ^c^ **<0,05**
Hip BMD (g/cm^2^)	0,746 ± 0,13	0,838 ± 0,109	0,985 ± 0,147	^ac^ ^b^ **<0,05**

Chi square test; *BMD* bone mineral density, *BMI* body mass index, *HDL* high density lipoprotein, *LDL* low density lipoprotein, *PAI 1* plasminogen activator inhibitor 1. Bolded p values are significant.

^a^osteoporosis group vs. normal BMD group; ^b^osteopenia vs. normal BMD group; ^c^osteoporosis vs. osteopenia group

### Associations of PAI-1 levels with metabolic parameters, BMD and bone turnover markers

PAI-1 positively correlated with lumbar BMD (*p* < 0,05) and proximal femur BMD (*p* < 0,000) as well as BMI (*p* < 0,000), insulin (*p* < 0,000) and triglycerides (*p* < 0,000) while negative correlation was observed in relation to age (*p* < 0,01), HDL cholesterol (*p* < 0,001), diabetes duration (*p* < 0,002), beta cross laps (*p* < 0,002), pyrilinks (*p* < 0,002) and osteocalcin (*p* < 0,002) (Table 2). The strongest explanatory variables for PAI-1 in the multiple regression analysis after adjusting for age and BMI among metabolic parameters were insulin and triglycerides levels as well as duration of diabetes. With the inclusion of drugs, only beta blocker (BB) and angiotensin convertase enzyme inhibitors/angiotensin receptor blockers (ACEi/ARB) therapy significantly correlated with PAI-1 levels, while there was no correlation with statin and metformin (Additional file 1: Table S2).

**Table 2:** Associations of PAI-1 levels and metabolic parameters, BMD and bone turnover markers; association of L-BMD and metabolic parameters, PAI-1 and bone turnover markers

PAI-1	*r*	*p*	L-BMD	*r*	*p*
Age (year)	−0,2375	0,011	Age (year)		
BMI (kg/m^2^)	0,3453	0,000	BMI (kg/m^2^)	0,347	0,000
Diabetes duration (year)	-0,3046	0,002	Diabetes duration (year)	0,297	0,000
Insulin (mIU/L)	0,4411	0,000	Insulin (mIU/L)	−0,232	0,018
Triglycerides (mmol/L)	0,4704	0,000	Triglycerides (mmol/L)		
HDL (mmol/L)	-0,3331	0,001	LDL (mmol/L)	-0,1956	0,041
Osteocalcin (ng/ml)	-0,2976	0,002	PAI-1	0,119	0,046
Pyrilinks (nMPD/mMc)	-0,2897	0,002	Osteocalcin (ng/ml)	−0,318	0,001
Crosslaps (ng/ml)	-0,2955	0,002	Pyrilinks (nMPD/mMc)	−0,302	0,001
Lumbar BMD (g/cm^2^)	0,1990	0,046	Crosslaps (ng/ml)	−0,24	0,012
Hip BMD (g/cm^2^)	0,3530	0,000			

*PAI 1* plasminogen activator inhibitor 1, *BMD* bone mineral density, *BMI* body mass index, *HDL* high density lipoprotein

If we look at bone markers in the multiple regression analysis after adjusting for age and menopause duration, negative association of pyrilinks and PAI-1 was present. In addition, hip BMD positively correlated with PAI-1 activity (Additional file 1: Table S3).

In the stepwise forward regression analysis triglycerides, insulin, waist circumference, diabetes duration, pyrilinks and BB treatment remained significantly correlated with PAI-1 after adjusting for age and BMI, but the correlation between PAI-1 and hip BMD was no longer significant as was the case with the correlation between PAI-1 and ACEi/ARB therapy (Table 3). The positive association was observed between PAI-1 level and triglycerides, insulin, BMI and waist circumference as well as BB therapy while significant negative correlation was present between PAI-1 activity and pyrilinks. Therefore, the strongest determinants of PAI-1 activity were triglyceride and insulin levels followed by pyrilinks and explained 57,4 % of the variability of PAI-1.

**Table 3:** Stepward forward regression analysis of PAI-1 and metabolic parameters, bone mineral density and therapy

Dependent variable: PAI-1	Beta	*p*	adjusted *R* ^2^
Triglycerides (mmol/L)	0,314	0,0002	0,574
Insulin (mIU/L)	0,250	0,0033	
Pyrilinks (nMPD/mMc)	−0,308	0,0002	
BMI (kg/m^2^)	0,310	0,0035	
Waist circumference (cm)	−0,278	0,0097	
Age (years)	−0,364	0,0201	
Diabetes duration (years)	−0,187	0,0185	
Menopause duration (years)	0,283	0,0688	
Beta blockers (%)	0,224	0,0045	

*PAI-1* plasminogen activator inhibitor 1, *BMI* body mass indeks

### Associations of lumbar bone mineral density with metabolic parameters, PAI-1 and bone turnover markers

Association of BMD with metabolic parameters and bone turnover markers are shown in Table 2. There was significant correlation between L-BMD and insulin which remained significant after adjusting for age and BMI (Table 4.A). L-BMD correlated positively with PAI-1, but after adjusting for age and BMI the association between L-BMD and PAI-1 did not remain significant. The strongest determinants of L-BMD were osteocalcin and insulin levels (Table 4.A).

**Table 4:** Stepward forward regression analysis of lumbar BMD and metabolic parameters (A); stepward forward regression analysis of pyrilinks and BMD and metabolic variables (B)

A: Dependent variable L-BMD	Beta	p	B: Dependent variable Pyrilinks	Beta	p
Age (years)	0,716	0,0002			
Menopause duration (years)	−0,718	0,016			
BMI (kg/m^2^)	0,279	0,0021		0,309	0,053
Waist circumference (cm)				−0,381	0,011
Triglycerides (mmol/L)					
Insulin (mIU/L)	−0,273	0,009			
PAI-1(U/l)	0,171	0,117		−0,274	0,0126
Pyrilinks (nMPD/mMc)					
Osteocalcin (ng/ml)	−0,391	0,0001			
L-BMD (g/cm^2^)				−0,319	0,0025
adjusted R^2^	0,408	0,00000	adjusted R^2^	0,251	0,0001

*PAI-1* plasminogen activator inhibitor 1, *BMI* body mass index, *L-BMD* lumbar bone mineral density

### Associations of bone turn over markers with metabolic parameters, BMD and PAI-1

Forward stepwise regression analysis was performed to determine the strongest predictors of pyrilinks. The strongest predictor of pyrilinks was L-BMD, followed by waist circumference (Table 4.B). The association between pyrilinks and PAI-1 remained significant after adjusting for age and BMI (*p* = 0,0126).

Forward stepwise regression analysis was performed to determine the strongest predictors of osteocalcin. When osteocalcin was entered as a dependent variable and waist circumference, diabetes duration, menopause duration, insulin, triglycerides, femoral and lumbar BMD, and therapy, as independent variables, the strongest predictors of osteocalcin were L-BMD, insulin and pyrilinks, *R*^2^ = 0,420, *p* = 0,000001 (data not shown).

## Discussion

It is generally considered that diabetes type 2 patients have higher BMD compared to non-diabetic control subjects [15], however, that particular population of patients also has increased risk of fractures [16]. This could be due to low grade inflammation which is more severe in subjects with high insulin resistance than in those with low insulin resistance [17, 18], presumably leading to lower quality of bones causing higher incidence of fractures despite normal BMD in diabetes type 2 patients. Therefore, a role of obesity and chronic inflammation in diabetes type 2 still remains elusive. Our research demonstrated that postmenopausal female patients with diabetes type 2 and normal BMD had signficantly lower bone turnover markers and higher BMI compared to diabetic subjects with osteoporosis/osteopenia indicating lower rate of bone turnover in obese subjects. In addition to the well established role of inflammatory cytokines in promoting enhanced osteoclast function and bone loss, proinflammatory cytokines may also inhibit osteoblast function and bone repair [5]. Whereas previous work suggests that osteoporosis is linked to inflammation, it is not yet clear whether higher CRP levels are associated with bone loss [19]. In recently published study CRP was associated inversely with composite strength index but not associated with femoral neck or lumbar spine BMD [20] which is consistent with our observation.

There is growing evidence that disturbances in production of plasminogen activator inhibitor 1, one of the pro-inflammation markers, could be responsible for lower bone quality in diabetic patients. Over-expression of PAI-1 in transgenic mice resulted in increased mineralization and biomechanical properties of mouse femora [12, 13]. However, there are no studies investigating a role of PAI-1 in human subjects with osteoporosis and diabetes type 2. If we look at PAI-1 levels in our study, a decreased concentration was observed in diabetic female subjects with lower bone mineral density (the difference being greatest between osteoporosis and normal BMD group) along with positive correlation with lumbar and femoral BMD and negative one with bone turnover markers, suggesting that increased leves of PAI-1 could protect against bone loss in diabetes type 2 female patients. However, if that assumption is true, then why are PAI-1 levels decreased in diabetic patients with osteoporosis compared to those with normal BMD? We can only speculate, that lower levels of PAI-1 were due to lower BMI. However, insulin levels were not significantly lower in diabetic patients with osteoporosis, possibly due to a relatively small number of subjects but consistently significant positive correlation was found between insulin and PAI after adjustment for age and BMI.

This notion is supported with previous findings where increased levels of PAI-1 were present in the insulin resistance syndrome, and a significant correlation was found between plasma PAI-1 levels and body mass index, triglyceride levels, insulin levels and systolic blood pressure [6, 7]. The possible mechanisms are still unclear and in-vitro data indicate that PAI-1 synthesis by endothelial cells and hepatocytes could be affected by insulin, proinsulin and atherogenic lipoproteins [21]. In our study, in the final regression analysis triglycerides, insulin, BMI and waist circumference significantly correlated with PAI-1 levels, whereas correlation of PAI-1 and BMD did not exist. In addition, bone turnover markers accounted for only 16 % of variance in PAI-1 levels, while metabolic parameters accounted for 39 % of total variance in the multiple regression analysis, implying that the influence of bone markers on PAI-1 levels was weaker compared to metabolic parameters. These results suggest that PAI-1 correlated primarily with metabolic parameters such as hyperinsulinemia, hypertriglyceridemia, obesity and adipose distribution, while association of PAI-1 and BMD and bone turnover was weaker. After adjustment for age and BMI, the association between PAI and both lumbar BMD and hip BMD was no longer significant, suggesting that metabolic parameters may affect both BMD and PAI-levels. Therefore, the association of PAI-1 and lumbar BMD could be only indirect. However, one of the bone resorption markers (pyrilinks) was independently and significantly negatively associated with PAI-1. Obviously, interaction between bone, body composition, endocrine, and inflammatory systems is complex and the underlaying mechanism of PAI influence on the bone remains unclear. This effect could be mediated through protective mechanisms of obesity and hyperinsulinemia in diabetes type 2 on bone mass, since the strongest determinants of circulating levles of PAI-1 were insulin and tryglicerides leveles, thus prevailing over deleterious effect of chronic inflammation on bone metabolism (4,5).

Postmenopausal women included in this study were all long term diabetes type 2 patients receiving standard care according to ADA recommendations [14]. Since majority of those medications could alter PAI-1 levels, the possible effect of drugs was also investigated. It is a known fact that lowering PAI-1 levels may be achieved by diet and/or exercise or by treatment with thiazolidinediones, metformin, statins or inhibitors of the renin–angiotensin system [22]. However, our data did not confirm those results. In the regression analysis model, only beta blocker therapy and ACEi/ARB significantly correlated with PAI-1 levels, while there was no correlation with statin and metformin therapy as was expected. This discrepancy could be explained with small sample size to obtain statistical significance, although according to previous studies, beta blockers have prothrombotic effect, and in fact increase PAI level [23] which is consistent with our results. In this study, PAI levels were not affected by statins nor metformin with the exception of beta blockers and ACEi/ARB, but usage of these drugs were not randomized. This supports the notion that various drugs have effect on circulating PAI-1 levels, however this is a real life study and an indicator for further research that the influence of therapy should not be ignored.

Our study has some limitations. A first limitation of our study is the cross-sectional design, which limits the assessment of causal relationships. A second limitation is the relatively small sample size of diabetic patients with respect to the complex questions addressed, especially after stratification according to BMD in three groups. Furthermore, this article does not assess fracture risk which is especially important in diabetic patients who presumably have lower quality of bone in spite of higher BMD as was stated previously [15, 16].

## Conclusion

In conclusion, this a first study investigating a role of PAI-1 in human subjects with osteoporosis and diabetes type 2. Our findings suggest that elevated PAI-1 levels are associated with higher BMD in obese diabetic patients but the possible implications of this finding and underlaying mechanisms still remain unclear. Obviously influence of PAI is primarily mediated through metabolic factors, hyperinsulinemia, hypertriglyceridemia, and obesity. However, the fact that pyrilinks also independently correlates to PAI-1 may suggest its direct involvement in bone metabolism influencing bone mass and strength. In conclusion PAI-1 appears to be a marker of metabolic status that correlates with measures of bone density and turnover; whether it is itself an agent that affects bone would need to be investigated in further studies.

## Availability of data and materials

The dataset supporting the conclusions of this article is included within the article (and its Additional file 2).

## Additional files:

Additional file 1: Tables S1, S2, S3. (docx 17 kb) Additional file 2: DM2_postmenopausis. (xlsx 88 kb)

## Abbreviations

ACEi/ARB: angiotensin convertase enzyme inhibitors/angiotensin receptor blockers
BB: beta blocker
B-CTx: beta-CrossLaps
BMD: bone mineral density
DXA: dual-energy x-ray absorptiometry
L-BMD: lumbar spine BMD measurements
PAI-1: plasminogen activator inhibitor-1
PYD: urinary pyrilinks-D
