# Supplementary material for: Plasminogen activator inhibitor-1 concentrations and bone mineral density in postmenopausal women with type 2 diabetes mellitus
Source: BMC Endocr Disord. 2016 Mar 3;16:14. doi: 10.1186/s12902-016-0094-x (PMC4778311; doi:10.1186/s12902-016-0094-x)
Supplement: Additional file 1: — Tables S1, S2, S3. (docx 17 kb) [file 12902_2016_94_MOESM1_ESM.docx]

**Table S1**. Risk factors and therapy of postmenopausal women with T2DM

|  | **Osteoporosis** | **Osteopenia** | **Normal BMD** |
| --- | --- | --- | --- |
| **Hypertension n (%)** | 17 (85) | 33 (76,6) | 36 (85,7) |
| **ACEi/ARB n (%)** | 9 (47,4) | 27 (71) | 27 (65,8) |
| **Beta blocker n (%)** | 3 (16,7) | 11 (29,7) | 16 (42.1) |
| **Statins n %** | 9 (42,9) | 29 (64,4) | 31 (68,9) |
| **Metformin n (%)** | 9 (42,9) | 21 (44,6) | 22 (44,9) |
| **Sulfonylurea n (%)** | 17 (80,9) | 39 (82,9) | 42 (85,7) |
| **DPP-4i n (%)** | 9 (42,8) | 18 (38,3) | 25 (51) |
| **monotherapy** | 7 (33,3) | 20 (42,5) | 15 (30,6) |
| **Two drug combination n (%)** | 4 (19,1) | 8 (17,0) | 10 (20,4) |
| **Three drug combinationa n (%)** | 9 (42,8) | 18 (38,3) | 22 (44,9) |
| **Nonsmokers n %** | 16 (80) | 33 (91) | 34 (87) |
| **Alcohol no n %** | 21 (100) | 45 (97) | 46 (97) |
| **HRT n%** | 3 (15) | 6 (12,7) | 10 (20,4) |

ACEi/ARB, angiotensin convertase enzyme inhibitor /angiotensin receptor blocker, DPP-4i, dipeptyl peptidase-4 inhibitors, HRT, hormone replacement therapy

**Table S2**. The strongest explanatory variables for PAI-1 among metabolic parameters determined by multiple regression analysis

|  |  |  |  |  |
| --- | --- | --- | --- | --- |
| **Model** | **Variable** | **Beta p** | **adjusted R^2^** |  |
| I | age  BMI  Diabetes duration  Insulin | 0,191 0,03  -0,187 0,025  0,235 0,012 | 0,321 |  |
| II | age  BMI  Diabetes duration  Insulin  Triglycerides | -0,195 0,016  0,229 0,01  0,302 0,0007 | 0,399 |  |
| III | age  BMI  Diabetes duration  Insulin  Triglycerides  Lumbar BMD | -0.210 0,011  0.235 0,009  0.308 0,0007 | 0.392 |  |
| IV | age  BMI  Diabetes duration  Insulin  Triglycerides  Hip BMD | -0.199 0,014  0.227 0,011  0.302 0,007 | 0.387 |  |
| V | age  BMI  Diabetes duration  Insulin  Triglycerides  Beta blockers | -0.208 0,015  0.214 0,024  0.302 0,0014  0.230 0,006 | 0.431 |  |
| VI | age  BMI  Diabetes duration  Insulin  Triglycerides  Beta blockers  ACEi/ARB  statins | 0.205 0,02  0.196 0,04  0.288 0,0025  0.213 0,014 | 0.429 |  |

BMD, bone mineral density; BMI, body mass index; ACEi/ARB, angiotensin convertase enzyme inhibitor /angiotensin receptor blocker.

**Table S3**. The strongest explanatory variables for PAI-1 among bone markers determined by multiple regression analysis

| **model** | **variable** | **Beta p** | adjusted **R²** |
| --- | --- | --- | --- |
| I | Osteocalcin  Pyrilinks D  Age | -0.214 0.036  -0.195 0.037 | 0.130 |
| II | Osteocalcin  Pyrilinks D  Age  Menopause duration | -0.258 0.022  -0.415 0.040 | 0.133 |
| III | Osteocalcin  Pyrilinks D  Age  Menopause duration  Lumbar BMD | **-**0.245 0.03  -0.435 0.033 | 0.131 |
| IV | Osteocalcin  Pyrilinks D  Age  Menopause duration  Hip BMD | -0.219 0.05  -0.376 0.06  0.228 0.043 | 0.162 |

BMD, bone mineral density
